# Effect of Processing on Postprandial Glycemic Response and Consumer Acceptability of Lentil-Containing Food Items

**DOI:** 10.3390/foods7050076

**Published:** 2018-05-11

**Authors:** D. Dan Ramdath, Thomas M. S. Wolever, Yaw Chris Siow, Donna Ryland, Aileen Hawke, Carla Taylor, Peter Zahradka, Michel Aliani

**Affiliations:** 1 Guelph Research and Development Centre, Agriculture and Agri-Food Canada, Guelph, ON N1G 5C9, Canada; Aileen.Hawke@AGR.GC.CA; 2 Glycemic Index Labs, Inc., Toronto, ON M5C 2N8, Canada; TWolever@gilabs.com; 3 Agriculture and Agri-Food Canada, St. Boniface Albrechtsen Research Centre, Winnipeg, MB R2H 2A6, Canada; YawChris.Siow@AGR.GC.CA; 4 Department of Food and Human Nutritional Sciences, University of Manitoba, Winnipeg, MB R3T 2N2, Canada; Donna.Ryland@umanitoba.ca (D.R.); Carla.Taylor@umanitoba.ca (C.T.); pzahradka@sbrc.ca (P.Z.); 5 Department of Physiology and Pathophysiology, University of Manitoba, Winnipeg, MB R3E 0J9, Canada; 6 Canadian Centre for Agri-food Research in Health and Medicine, Winnipeg, MB R2H 2A6, Canada

**Keywords:** lentil, processing, glycemic response, glycemic index, human trial, acceptability

## Abstract

The consumption of pulses is associated with many health benefits. This study assessed post-prandial blood glucose response (PPBG) and the acceptability of food items containing green lentils. In human trials we: (i) defined processing methods (boiling, pureeing, freezing, roasting, spray-drying) that preserve the PPBG-lowering feature of lentils; (ii) used an appropriate processing method to prepare lentil food items, and compared the PPBG and relative glycemic responses (RGR) of lentil and control foods; and (iii) conducted consumer acceptability of the lentil foods. Eight food items were formulated from either whole lentil puree (test) or instant potato (control). In separate PPBG studies, participants consumed fixed amounts of available carbohydrates from test foods, control foods, or a white bread standard. Finger prick blood samples were obtained at 0, 15, 30, 45, 60, 90, and 120 min after the first bite, analyzed for glucose, and used to calculate incremental area under the blood glucose response curve and RGR; glycemic index (GI) was measured only for processed lentils. Mean GI (± standard error of the mean) of processed lentils ranged from 25 ± 3 (boiled) to 66 ± 6 (spray-dried); the GI of spray-dried lentils was significantly (*p* < 0.05) higher than boiled, pureed, or roasted lentil. Overall, lentil-based food items all elicited significantly lower RGR compared to potato-based items (40 ± 3 vs. 73 ± 3%; *p* < 0.001). Apricot chicken, chicken pot pie, and lemony parsley soup had the highest overall acceptability corresponding to “like slightly” to “like moderately”. Processing influenced the PPBG of lentils, but food items formulated from lentil puree significantly attenuated PPBG. Formulation was associated with significant differences in sensory attributes.

## 1. Introduction

Lentils (*Lens culinaris* L.), along with other dry edible varieties of beans and peas, are referred to as pulses; they are rich in protein and complex carbohydrates, and make important contributions to micronutrient intake [1,2]. Currently, there is a growing body of evidence that suggests the regular consumption of lentils, along with other pulses, is associated with reduced risk for cardiovascular disease and type 2 diabetes [3,4,5,6], as well as improved glycemic control and lowering of blood pressure [7,8]. There is also evidence in support of the quantitative health benefits of pulses; Kabagambe estimated that the daily consumption of one serving of beans (~86 g) per day is associated with 38% reduction in risk for myocardial infarction [3]. These findings have stimulated renewed interest by the pulse industry and healthcare providers to promote the increased consumption of pulses. Such efforts could be enhanced with regulatory approval of an appropriate pulse health claim; however, attempts to achieve this have been hampered by a lack of sufficient high-quality human clinical trials.

Many of the human studies on pulses have been criticized for being under-powered and conducted with relatively small samples and poorly characterized food products. Further, structure-function studies are required in order to identify the active components and understand the mechanism by which pulses confer their health benefits. Lentils and other pulses contain useful amounts of bioactives such as polyphenols, fatty acids, carotenoids, and antioxidants [9]. Polyphenol extracts from different lentil varieties exhibit significant in vitro inhibition of α-glucosidase, a major intestinal enzyme involved in the digestion of carbohydrates [10,11], and may provide a partial explanation for the low post-prandial blood glucose response (PPBG) associated with lentils [12]. 

In an attempt to increase consumption, lentils and other pulses are being milled into flours and incorporated into food products [13,14,15,16,17,18], with the assumption that pulses will maintain their ability to reduce PPBG after being processed. Cryne et al. (2012) found that spray-dried pulse consumption did not affect cardiovascular disease risk or glycemic control [19], whereas Anderson et al. (2014) concluded that commercially available pulse powders maintain their ability to reduce PPBG in healthy young men [20]. Using whole yellow pea flour, Marinangeli et al. (2009) formulated different novel functional foods and found that banana bread and biscotti, but not pasta, produced lower glycemic responses compared with white bread [16]. In the case of lentils, Jenkins et al. (1982) found that boiling (for 20 or 40 min) resulted in a flattened PPBG in human volunteers; this was unchanged by blending lentils to a paste [21]. However, when the boiled and blended lentils were dried (12 h at 250 °C), an approximate three-fold increase in PPBG was observed. Collectively, these studies highlight the need for high-quality structure-function human trials using well-characterized intervention foods.

To support a potential health claim for PPBG-lowering, a long-term randomized controlled human trial with lentil- (test) and potato-based (control) foods would be required. However, prior to undertaking such a trial, it is necessary to ensure that the test and control foods not only possessed the desired difference in PPBG, but were also acceptable to participants so as to ensure compliance with the study diets. It was recognized that the efficacy of the intervention foods and consumer acceptability may be affected by the processing methods used to obtain lentils in formats that are suitable for the food matrices being considered. Therefore, the purpose of this study was: (i) to determine the glycemic index (GI), PPBG, and relative glycemic responses (RGR) of lentils subjected to different processing techniques; and (ii) to use these results to formulate food items containing lentils and assess the PPBG, RGR, and consumer acceptability of these foods. Since the planned intervention trial also required control products, food items containing instant potato flakes, a high-GI food, instead of lentils were also studied for their effects on PPBG and RGR.

## 2. Methods

### 2.1. Lentil Processing

Large green lentils (var CDC Greenland, Canadian Certified #1) from the 2010 crop year (Simpson Seeds Inc., Moose Jaw, SK, Canada) were kindly provided by Alliance Grain Traders Inc. (Regina, SK, Canada) and processed in order to obtain different starting materials for food development. Fixed amounts of raw lentils were rinsed then cooked in filtered water (1:2.5 (*wt*:*wt*) ratio) by bringing to a boil then simmering for 36 min at medium heat, stirring, and allowing to sit for 10 min; this was designated boiled whole lentils (BWL). Boiled lentil puree (BLP) was produced from BWL by adding 40 g of filtered water and grinding in a mini Cuisinart food processor to form a puree. Frozen boiled lentil puree (FLP) blocks were prepared from BWL, as above, and pureed (Stephan Model UM 12 grinder; Stephan Machinery Corporation, Manhasset, NY, USA) by adding a small quantity of water to obtain a smooth paste. The puree was dispensed in round silicone molds, shaken to remove air bubbles and frozen at −65 °C for 2 h (Kuler, Lutrafrost FG1301, Union Carbide Canada Ltd., Lachine, QC, Canada). The frozen blocks were quickly unmolded, packed as single units in plastic bags, vacuum sealed then stored at −40 °C until use. Roasted lentil flour (RLF) was prepared by spreading a thin layer of whole lentils in a rotating oven (Picard Model MT-4-8, Équipement de Boulangerie LP Inc., Victoriaville, QC, Canada) for 20 min at 120 °C. It was then cooled in a freezer before being ground into flour. Cooked spray-dried lentil flour (DLF) was prepared by first rinsing the whole lentils in demineralized water, cooking, and then mixing in a water jacketed bulk tank (35 kg/110 L of demineralized water) for a total of 30 min to a maximum temperature of 95 °C. The mixture was cooled then ground with three successive passes of a pump feed knife grinder (Stephan Mikrocut Type MC15, Stephan Machinery Corporation, Manhasset, NY, USA) using a 1.5-mm knife for the first pass, followed by a 0.2-mm knife for the next passes. Water (20 L) was added after the first pass, and then additional water was included after the last pass to obtain a Brix solution at 5.7 °C. The mix was spray-dried using an atomizer model Type HT 10-530 (Niro Atomizer Ltd., Copenhagen, Denmark) set with inlet and outlet air temperatures of 181 °C and 77 °C, and air flow rate around 25–40 psig.

### 2.2. Nutrient Analysis

Core proximate and dietary fiber analyses of all test foods were conducted by Maxxam Analytics (Mississauga, ON, Canada) using standard AOAC methods for total fat (AOAC 922.06, 933.05), ash (AOAC 923.03), protein (AOAC 992.15), moisture, and total dietary fiber (TDF; AOAC 991.43, 985.29). Energy and total carbohydrates were derived, as was available carbohydrate (avCHO = total carbohydrates − TDF). Proximate and dietary fiber analyses of white bread (WB) were provided by Glycemic Index Laboratories (Toronto, ON, Canada).

### 2.3. Preparation of Food Items from Lentil and Potato

Boiled lentil puree was used as the primary source of carbohydrate during the formulation of lentil food items; these were standardized at 25 g avCHO in order to provide a serving that could be easily consumed within 10 min. Control foods were prepared using Instant Potato Flakes (IPF: Idahoan Original Mashed Potatoes, Idahoan Foods^©^ 2008, Lewisville, ID, USA) and standardized at 50 g avCHO. The formulation breakdown, processing time, and temperatures of items prepared with IPF or BLP are summarized in Table 1.

### 2.4. Glycemic Response and GI Studies

Three acute human feeding trials were conducted: Study 1 assessed the glycemic response, GI, and RGR of cooked whole lentil products; Study 2 assessed the glycemic response and RGR of food items containing BLP, which was the primary source of carbohydrate; Study 3 assessed the glycemic response and RGR of mixed meals in which IPF was the primary source of carbohydrate. Studies 2 and 3 ran in parallel. All study protocols were approved by the Western Institutional Review Board (Puyallup, WA, USA; Protocol #441 WIRB) and informed consent was obtained from all participants for each series of tests.

### 2.5. Screening of Participants

Prior to their participation, volunteers were screened to assess eligibility. Males or non-pregnant females between the ages of 18 and 75 years with a body mass index (BMI) < 30 kg/m^2^ and normal fasting serum glucose (<7.0 mmol/L capillary corresponding to whole blood glucose < 6.3 mmol/L) who were willing to maintain habitual diet, physical activity pattern, and body weight throughout the trial, understood the study procedures, and were willing to provide informed consent were eligible to participate in the study. Participants were excluded from participation if they met any of the following exclusion criteria: known history of HIV/AIDS, hepatitis, diabetes, or a heart condition; gastrointestinal disease; regular consumption of supplements which have an effect on blood glucose response; tobacco use; known intolerance, sensitivity or allergy to any ingredients in the study products; other medical, psychiatric, or behavioral factors that in the judgment of the Principal Investigator may interfere with study participation or the ability to follow the intervention protocol; shift workers; major trauma or surgical event within 3 months of screening; unwillingness or inability to comply with the experimental procedures and to follow safety guidelines; failure to meet any one of the inclusion criteria.

A pool of 20 eligible participants was recruited to participate in the three glycemic response studies. Each study included *n* = 10 participants, all of whom completed the study. Two participants completed all three studies; one completed Studies 1 and 2; three completed Studies 1 and 3; and two completed Studies 2 and 3.

### 2.6. Statistical Power

Based on the t-distribution and assuming an average coefficient of variation for the within-individual variation of incremental area under the blood glucose response curve (iAUC) values of 25%, *n* = 10 participants provided 80% power to detect a 35% difference in iAUC with a two-tailed *p* < 0.05.

### 2.7. Study Procedures

Each study followed an open-label, randomized, cross-over design. Participants willing to be considered were invited to the clinical trial site (Glycemic Index Laboratories Inc., Toronto, ON, Canada) to have the study procedures explained and be given a copy of the consent form. They were encouraged to ask questions and to not sign the consent form until all of their questions were answered. Those who agreed to participate were asked to return to the trial site for a pre-selection visit when eligibility was determined from responses to a screening questionnaire, and results of their BMI and fasting blood glucose measurements. Eligible participants were studied on 8 to 10 separate days over a period of 4 to 10 weeks. The interval between successive tests was no less than 48 h and no more than 2 weeks. Participants were asked to maintain stable dietary and activity habits throughout their participation in the study, as well as to refrain from drinking alcohol and from unusual levels of food intake or physical activity for 24 h before each test. Any participant who was unwell or had not complied with the preceding experimental conditions was rescheduled for testing. On each test day, participants arrived at the trial site in the morning after a 10–14 h overnight fast. They were first weighed and two fasting blood samples for glucose analysis (2–3 drops into a fluoro-oxalate tube) were obtained by finger-prick 5 min apart. After the second fasting blood sample, the participant started to consume a test meal along with a drink of one or two cups of water, coffee or tea with 30 mL 2% milk per cup, if desired. The drink chosen remained constant for all test foods. At the first bite a timer was started and additional blood samples for glucose analysis (2–3 drops into a fluoro-oxalate tube) were taken at 15, 30, 45, 60, 90, and 120 min. Participants’ hands were warmed with an electric heating pad for 3–5 min prior to each blood sample. During the test, participants remain seated quietly and were asked to record any unusual activities on the previous day. After the last blood sample, participants were offered a snack and were allowed to leave. Glucose analysis was performed with a YSI model 2300 STAT analyzer (Yellow Springs, OH, USA). Blood glucose in the 0 min sample was measured in duplicate to allow an estimation of analytical precision.

### 2.8. Glycemic Response in Study 1 

The participants in this study were four males and six females aged (mean ± standard deviation (SD)) 40 ± 10 years with BMI 25.0 ± 4.1 kg/m^2^. The serving size for all test and control foods was set at 50 g avCHO, based on direct composition analysis (Table 2). The whole lentil products tested were: BWL, BLP, FLP, RLF, and DLF. In addition, participants consumed IPF once, as a positive control, and the reference white bread (WB) on two separate occasions. The whole lentil products were prepared as described above, with BWL and BLP being prepared on each test day. FLP was prepared by thawing five frozen blocks in a microwave oven at high setting for approximately 3 min, stirring to mix, and then heating for an additional 2 min. RLF, DLF, and IPF were prepared by adding 300 g, 290 g, and 372 g of filtered boiling water, respectively. After mixing, the lentil products were microwaved for 30 s and IPF for 1 min. WB was baked by Glycemic Index Laboratories in an automatic bread maker using weighed ingredients. In this study, all lentil and potato test foods were served with 10 g tomato juice (Heinz brand) and 0.05 g oregano to improve palatability. 

### 2.9. Glycemic Response in Study 2

The participants in this study were four males and five females aged (mean ± SD) 51 ± 10 years with BMI 26.2 ± 4.6 kg/m^2^. In order to achieve a food portion that could be easily consumed within 10 min, the serving size for all test and control foods was set at 25 g avCHO, based on direct composition analysis (Table 3). The test foods were all made with BLP and included: Apricot Chicken (AC); Chicken Pot Pie (CPP); Lemony Parsley Soup (LPS); Lentil Soup (S); Side Dish (SD); Minestrone Casserole (MC); Meatloaf (ML); Vegetarian Meatloaf (VM). The reference white bread was consumed on two separate occasions. The test foods were prepared, as outlined in Table 1, at the Weston Sensory and Food Research Center, University of Manitoba, Winnipeg. They were stored frozen and shipped in batches to the trial site. On the morning of testing, food items were defrosted and the portion was weighed out and heated in a microwave oven before serving to the study participants.

### 2.10. Glycemic Response in Study 3

The participants in this study were four males and five females aged (mean ± SD) 41 ± 11 years with BMI 24.4 ± 2.6 kg/m^2^. The serving size for all test and control foods in Study 3 was set at 50 g avCHO, based on compositional analysis (Table 3). These foods items were made with IPF and formulated to match the food matrix of the various lentil food items: Apricot Chicken (pAC); Chicken Pot Pie (pCPP); Lemony Parsley Soup (pLPS); Potato Soup (pS); Vegetable Casserole (pSD); Minestrone Casserole (pMC); Sheppard’s Pie (pSP); Vegetarian Meatloaf (pVM). The reference white bread was consumed on two separate occasions. The test foods were prepared and shipped as outlined above. As before, on the morning of testing, food items were defrosted and the portion was weighed out and heated in a microwave oven before being served to study participants.

### 2.11. Consumer Acceptability Studies

Selected foods (AC, CPP, LPS, LS, MC, ML) containing 132 g (0.6 cup) of whole cooked lentils to be used for the planned 12-week randomized control human trial were assessed for consumer (*n* = 92) acceptability. The selected foods were prepared in quantities sufficient for the consumer study and frozen (−20 °C) in plastic freezer bags (17.7 × 18.8 cm) (Ziploc, S. C. Johnson and Son Limited, Brantford, ON, Canada) for 5 to 7 weeks. The test foods were removed from the freezer approximately 16 to 18 h prior to serving and placed at 4 °C. The four side dishes, AC, CPP, MC, and ML (40 g for each), were portioned into aluminum foil tart tins 2 cm high with a top interior diameter of 7.5 cm and a bottom diameter of 5 cm (Bake King, Novelis Inc. Montreal, QC, Canada) and covered with a custom-made pear shaped aluminum foil cover. Samples were baked (Frigidaire Professional Series Even Cook conventional oven, Electrolux Canada Corp., Mississauga, ON, Canada) on metal trays at 150 °C until an internal temperature of 76 ± 2 °C was reached. To maintain temperature while samples were evaluated, the four tins were placed on one hot plate (Corning, Model PC-300, New York, NY, USA) set at 50 °C. Two soup samples, LPS and LS (50 g each), were heated on the range top of the appliance noted above in 2-L saucepans over medium heat to 85 ± 2 °C and were placed in 125-mL Styrofoam cups and covered (Dixie, Georgia-Pacific, Atlanta, GA, USA) immediately prior to serving to consumers.

### 2.12. Consumer Recruitment 

Volunteers were recruited from the staff and student populations according to procedures approved by the Joint-Faculty Human Ethics Research Board at the University of Manitoba (Protocol J2010:082). Criteria for study participants included availability, interest in volunteering, and no allergies to any food products. An honorarium was provided.

### 2.13. Evaluation of Test Foods

Consumers were seated in individual partitioned booths with overhead fluorescent lighting. Questionnaires to assess acceptability were administered and responses entered using computerized sensory software (Sensory Information Management Software, 2011). Filtered water (22 °C) was available for cleansing the palate as required. All consumers evaluated the six food samples during one session. The samples were labeled with different three-digit random numbers and presented in a completely randomized order. After looking at and tasting as much of the sample as desired, consumers rated their acceptance of aroma, appearance, flavor, and texture as well as overall acceptance on nine-point hedonic scales [22]. As another measure of acceptance, the Food Action rating scale (FACT) was also used [23]. Consumers were asked how often they would eat the lentil product and selected from one of the following nine options where 9 = “I would eat this every opportunity I had”; 8 = “I would eat this very often”; 7 = “I would frequently eat this”; 6 = “I like this and would eat it now and then”; 5 = “I would eat this if available but would not go out of my way”; 4 = “I do not like this but would eat it on an occasion”; 3 = “I would hardly ever eat this”; 2 = “I would eat this if there were no other food choices”; 1 = “I would eat this only if forced”. Information on gender, age, and frequency of eating lentils was also collected using a questionnaire. 

### 2.14. Calculations and Data Analysis

For each glycemic response study, incremental areas under the glucose response curves (iAUC), ignoring areas below fasting, were calculated [24], with fasting blood glucose taken to be the average of the values measured at −5 min and the first measurement at 0 min. For Study 1, the GI of the foods was calculated by expressing the iAUC for each test food as a percentage of the mean iAUC elicited by white bread in the same participant and multiplying the resulting values by 0.71 to convert to the glucose scale (i.e., the GI of glucose = 100). In Studies 2 and 3, RGR was calculated by expressing the iAUC for each test food as a percentage of the mean iAUC elicited by white bread in the same participant. Although RGR is calculated in the same way as GI, it was termed RGR because most of the test meals in Studies 2 and 3 consisted of a mixture of carbohydrate foods plus sources of fat and protein. GI is a property of individual carbohydrate foods, whereas the glycemic response elicited by mixed meals is influenced not only by the carbohydrates but also the fat and protein they contain [25]. However, since the effects of fat and protein on glycemic responses are similar in a variety of different participants [26], RGR can be used to compare the glycemic impact of the test meals in Studies 2 and 3. SigmaPlot for Windows (Ver. 13, Systat Software Inc., San Jose, CA, USA) and SPSS (Ver. 20, IBM SPSS Statistics, Inc., Armonk, NY, USA) were used for all statistical analyses with the level of significance set at *p* <  0.05. Treatment effects of foods on iAUC, RGR, and GI were determined using repeat measures ANOVA followed by Tukey’s post hoc test, where applicable, with the criterion for significance being two-tailed *p* < 0.01. All data for iAUC, RGR, and GI are reported as the mean ± standard error of the mean (SEM).

For consumer acceptability, four-way analysis of variance (ANOVA) was conducted with the Consumer (C) as a random effect and Formulation (F), Gender (G), and Age Group (A) as fixed effects. The two-way interactions of F*G and F*A were analyzed. Tukey’s multiple comparison test was used to determine differences among means when significant (*p* < 0.05). When interactions were not significant, sums of squares were pooled with the error and the F values for the remaining sources of variation for each variable were recalculated [27]. A principal component model (PCA) was fit where the value for overall acceptability for each consumer was plotted with consumers represented as vectors and samples represented by points on the graph. Statistical Analysis Software (SAS, Cary, IN, USA) was used for ANOVA and Tukey’s analysis, and XLSTAT (Addinsoft, Paris, France) were used for consumer internal preference mapping.

To visualize the overall acceptability of the six meals presented to the consumers in our study, the method of internal preference mapping was applied (XLSTAT, Addinsoft, Paris, France). This is a type of principal component model whereby the products are projected in the space that is defined by the judges. After the PCA step, the internal preference mapping tool removes the judges that are not adequately displayed on a two-dimensional space from the d-dimensional space. Communality (sum of squared cosines between the vector and the axis of the sub-space) is the measure used. All the retained judges are moved onto a virtual circle encompassing the product points, thus a true bi-plot is not represented. In the present study, the value for overall acceptability for each consumer was plotted with consumers represented as vectors and samples represented by points on the graph. Hence, the more vectors (consumers) found at a sample point, the more acceptable the product. 

## 3. Results

### 3.1. Glycemic Response Studies

A total of 22 test foods were assessed for their in vivo PPBG and RGR; GI was determined only on the whole lentil products. No statistical analysis was conducted on the nutritional composition of these foods, however, within each type of food there were obvious variations in fat, protein and total dietary fiber (Table 2 and Table 3), especially between foods containing lentil or potato. The average age, BMI, and male/female ratio of participants in Study 1 (40.0 ± 3.3 years; 25.0 ± 1.3 kg/m^2^; 4/6) and Study 2 (41.3 ± 3.5 years; 24.4 ± 0.8 kg/m^2^; 5/5) were not different. Participants in Study 3 (50.6 ± 3.2 years; 26.2 ± 1.4 kg/m^2^; 5/5) were slightly older. In terms of ethnicity, about half of the participants in each study were Caucasian and the other half comprised a mixture of South Asian and Chinese ethnicities. Overall, the coefficient of variation for blood glucose response elicited by the reference food, WB, as derived from the iAUC values for the repeated tests, was well below the acceptable limits of <30%.

**Figure 1:**
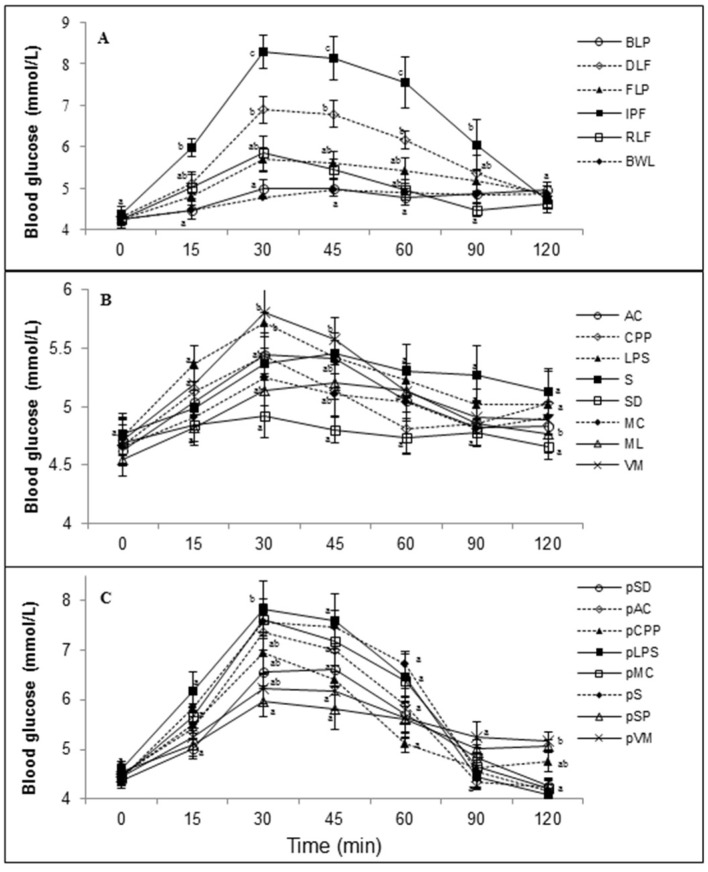
Blood glucose responses elicited by the different study foods. (**A**) Processed lentil products from Study 1: BWL, Boiled Cooked Lentil; BLP, Boiled Lentil Puree; FLP, Frozen Cooked Lentil Puree; RLF, Roasted Lentil Flour; DLF, Spray-Dried Lentil Flour; IPF, Instant Potato Flakes). (**B**) Food items containing pureed lentil from Study 2: AC, Apricot Chicken; CPP, Chicken Pot Pie; LPS, Lemony Parsley Soup; S, Lentil Soup; SD, Side Dish; MC, Minestrone Casserole; ML, Meatloaf; VM, Vegetarian Meatloaf. (**C**) Food items containing potato from Study 3: Pac, Apricot Chicken; pCPP, Chicken Pot Pie; pLPS, Lemony Parsley Soup; pS, Potato Soup; pSD, Vegetable Casserole; pMC, Minestrone Casserole; pSP, Sheppard’s Pie; pVM, Vegetarian Meatloaf. Values are expressed as means ± SEM for 10 participants; means with different letter superscripts differ significantly (*p* < 0.01, Tukey’s test) within a given time point.

Figure 1a shows that the blood glucose response of the processed whole lentil products and IPF tested in Study 1 differed significantly (*p* < 0.05) in magnitude and with time. Compared to BWL and FLP, DLF produced significantly higher blood glucose levels between 30 and 60 min (*p* < 0.05). In Study 2 (Figure 1b), after the consumption of food items made from lentil puree, the blood glucose response indicated significant treatment effects with the peak blood glucose at 30 min being significantly lower (*p* < 0.05) for SD compared to LPS and VM. At 45 min, VM had significantly higher (*p* < 0.05) blood glucose than SD. Figure 1c shows the blood glucose response for the food items prepared with potato in Study 3. At 30 min, blood glucose for pSP was significantly lower (*p* < 0.05) than pLPS. Unlike processed lentils and lentil food items, potato food items resulted in significant differences in blood glucose at 120 min: pSP and pVM values were higher (*p* < 0.05) than pLPS, pS, pMC, pAC, and pSD. 

Results of iAUC, RGR, and GI for processed lentils and food items showed a fair amount of heterogeneity within each category. The processed whole lentil products all had significantly lower (*p* < 0.05) mean iAUC than IPF, and DLF had a mean iAUC that was almost twice that of BWL, BLP, and RLF, but not significantly different from FLP (Table 2). Compared to IPF, the whole lentil products had significantly lower GIs (*p* < 0.05). Conversely, DLF had a mean GI that was significantly higher (*p* < 0.05) than the other lentil products. The palatability of the test foods was assessed by participants, and the least processed lentils (BWL and BLP) were ranked significantly higher than FLP and DLF; RLF was the least palatable (Table 2).

Within the lentil food items there were varying glycemic responses, with lentil side dish (SD) having a significantly lower (*p* < 0.05) mean iAUC than AC, LPS, S, ML, and VM (Table 3). Significant differences were also observed in iAUC among the potato food items, with the soup (pS) having a higher (*p* < 0.05) iAUC than most of the other meals. It was not meaningful to compare iAUC between lentil and potato food items since different amounts of avCHO were used in the two studies; instead, a comparison of RGR, standardized for white bread glycemic response, was made. Among the lentil food items, SD had the lowest RGR, which was significantly lower (*p* < 0.05) than AC, LPS, and VM. Among the different potato food items, PS had the highest RGR, which was significantly higher (*p* < 0.05) than pAC, pCPP, pSD, pSP, and pVM. Overall, lentil-based food items had a significantly lower mean RGR (*p* < 0.001) than those prepared from potato; the average difference in RGR was 32. Lentil side dish (SD) had an RGR that was significantly lower than all other lentil and potato mixed meals (*p* < 0.01). Among the potato mixed meals, potato soup (pS) stood out as having a significantly (*p* < 0.01) higher RGR than all of the lentil meals and most of the potato-based meals.

### 3.2. Consumer Demographics

Of the 92 consumers in this study, 72 were female. The age groups contained similar numbers: 18 to 24 years (*n* = 27); 25 to 34 years (*n* = 30); and ≥35 years (*n* = 35). The highest frequencies of eating lentils in any form, such as in soups, stews, side dishes, and casseroles, were “at least once a month” (26 %) and “a few times a year” (32%). About 10% stated that they had never eaten lentils. 

### 3.3. Sample Evaluation

A significant difference was shown for consumers for all attributes; this is to be expected, as everyone has their own acceptance criteria, methods for using the category scales, and sensitivities to the ingredients present in the lentil products (Table 4). Formulation was associated with significant differences for each attribute. There was, however, a significant interaction between gender and formulation for appearance. Although the number of males participating in the study was fewer than females, there could possibly be a trend toward higher acceptability of appearance for MC, AC, and LS by males compared to females. Appearance, flavor, and frequency of consuming the samples (food action rating scale—FACT) yielded differences between age groups. For appearance and flavor, the acceptability values were significantly higher in the oldest age group compared to the youngest age group. For FACT, the values were significantly higher for the oldest age group compared to both the younger groups. The formulation receiving the highest acceptability score for aroma was LPS, corresponding to “like moderately” (6.7), in contrast to MC which had the lowest aroma acceptability, corresponding to “neither like nor dislike” (5.4).

**Figure 2:**
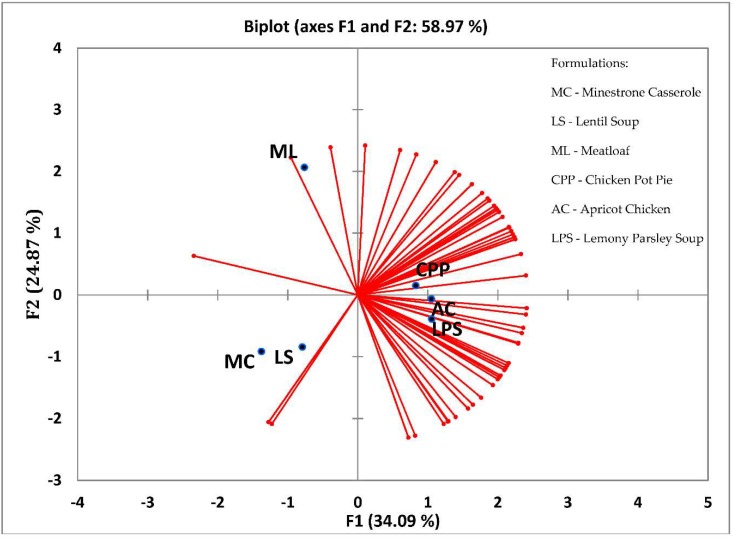
Consumer vector plot for overall acceptability.

The acceptability scores of the samples were in the same order for flavor, texture, and overall acceptability, ranging from approximately 7 (“like moderately”) to 5 (“neither like nor dislike”). AC, LPS, and CPP fell in the upper range (7.0 to 6.2), ML and LS in the mid-range (5.9 to 5.7), and MC in the lower range (5.6 to 5.0). These results are graphically represented in Figure 2. The majority of the vectors, which represent large numbers of consumers with high overall acceptability values, are shown grouped in an area on the right side near the x-axis of the graph, where AC, CPP, and LPS are also located. A small number of responses are shown in the area on the left side of the graph, where ML is located at the top, and LS and MC at the bottom. Since AC, CPP, and LPS were liked by more consumers than ML, LS, and MC, they would be recommended for further in-depth sensory analysis. 

## 4. Discussion

We used three well-designed, high-quality human trials to investigate the acute effect of processing on the PPBG-lowering action of lentils and quantified the relative reduction in PPBG and consumer acceptance of commonly consumed food items prepared from pureed whole green lentils. Acceptability is critical for compliance in clinical trials and for the possible further commercialization of lentil-containing foods [28]. Although there was some evidence that processing lentils affects PPBG, boiled whole lentils and boiled lentil puree appear to do so with equal effectiveness. Only DLF had significantly higher blood glucose responses compared to boiled whole, pureed, or roasted lentil flours. Boiled lentils appear to retain their beneficial effects on glucose response, as shown by others [29,30], and previous studies have also shown that grinding lentils before cooking leads to higher acute PPBG than boiled whole lentils [21,31]. In a long-term study (4 weeks), the consumption of spray-dried lentil powder had no significant effects on cholesterol, fasting glucose, or fecal endpoints [32] or on cardiovascular disease risk and glycemic control [19]. However, examination of the PPBG-lowering effect of roasted whole lentils and boiled lentil puree has not been previously reported. Clearly, knowledge of processing effects on lentils can contribute to the design of optimal intervention food products for use in human feeding trials that aim to assess the long-term effect of lentil consumption on glycemic control. 

The PPBG-lowering property of whole lentils has been attributed to the presence of higher amounts of dietary fiber, protein, and phenolic compounds [32]. It has also been suggested that the low glycemic response of lentils is linked to the presence of an intact cell wall, which serves as a barrier to starch gelatinization and enzymatic hydrolysis. As such, variations in the rate of pulse starch digestion is influenced by the extent to which pulse cell wall is disrupted, particle size, and changes in different starch fractions (e.g., resistant starch) during processing [31,33]. Results from the present study show that DLF contained substantially lower TDF than the other lentil flours; this may be attributed to a reduction in resistant starch content as a result of spray-drying. Consequently, the higher iAUC, RGR, and GI measured in DLF could be due to the smaller particle size of spray-dried flour [34] and the lower TDF, which together could reduce the physical barrier. O’Dea and Wong [35] showed that although ground and cooked lentils had a higher peak glucose response than boiled whole lentils, neither peak insulin nor insulin AUC was different. These authors concluded that the protein content of lentils, and not its viscosity nor the rate of intestinal starch hydrolysis, was likely to influence the metabolic response to legumes. One observation overlooked in that study is that peak glucose and insulin occurred earlier with ground lentils [35], which suggests that the rate of intestinal starch hydrolysis was in fact higher.

This study confirmed that cooked pureed lentils can be used instead of instant potato flakes to produce a range of food items with low iAUC, RGR, and GI, and with similar consistency and consumer acceptability under normal storage conditions. Previous studies on GI and glycemic response of food products prepared from pulse flour have produced inconsistent results. For example, Marinangeli et al. [16] reported a reduction in GI when wheat flour was replaced with whole yellow pea flour in banana bread and biscotti, but not pasta. Goni et al. [36] found that the GI of wheat pasta decreased from 73 to 58 when 25% of wheat was replaced with chickpea flour. On the other hand, Johnson et al. [37] found that the PPBG of a chickpea bread or an extruded chickpea bread was not significantly different from the white bread control. It is possible that these observations could be due to a low dose or physical changes in the pulse ingredients.

In the present study, differences in the pulse ingredient were minimized by using lentil puree as the common starting material, given that food matrix components such as dietary fiber [38], protein [39,40], fat [41], and anti-nutrients (phytic acid, phenols, and tannins) [7,42] can all influence intestinal starch digestion. Regardless, there were significant differences in iAUC and RGR among the lentil-containing food items and it is likely that these differences could be attributed to the presence of other ingredients. For example, lentil side dish (SD) was prepared with very few ingredients and had the lowest RGR among all of the lentil items. Other variables that may have contributed to the differences in iAUC and RGR include cooking method, cooking time, temperature of cooking, and the amount of water added to these meals. Previous work has shown that the cooking time of lentils is directly correlated with starch hydrolysis [43] and added water in the formulation plays a major role in starch gelatinization and subsequent blood glucose response [16]. Meals with similar RGR (AC vs. pAC, ML vs. pSP, and VM vs. pVM) had comparatively higher fat and protein content, suggesting that these two components may have influenced intestinal starch digestion and therefore played a role in influencing PPBG. Overall, considering all of the chemical and physical factors and their interactions, it is reasonable to expect that the food matrix has considerable influence on the glycemic response of mixed food items made from lentil puree. 

Regarding the acceptability of selected lentil-containing foods AC, CPP, and LPS had the highest overall acceptability corresponding to “like slightly” to “like moderately”, with acceptability of aroma, flavor, and texture also yielding the same results. It is recommended that a descriptive analysis sensory panel be conducted to determine which specific sensory attributes influenced the acceptability of these lentil-containing meals so that improvements can be made to the formulations with the aim of increasing acceptability. 

## 5. Conclusions

This study confirms that whole boiled or puree lentils are low GI foods, but spray-drying and milling lentils into a flour results in a significant change in blood glucose response; this was also affected by freezing boiled lentil puree, but to a lesser extent. Therefore, when incorporating lentils into food with the purpose of delivering low GI properties, a careful evaluation of the processing method and perhaps the storage conditions is warranted. In this study, a large green lentil variety was evaluated; however, a recent study showed that other lentil varieties (e.g., red lentil) have similar efficacies in lowering PPBG [44]. Although it is important to understand the reasons for variations in PPBG among different food types, it is clear that the reformulation of meals by replacing high glycemic food ingredients like potato with lentil is a useful approach for managing persons with impaired glycemic control and promoting healthy eating [6]. Future studies should be conducted to determine whether the replacement of other high-GI food ingredients (e.g., wheat flour or rice) with lentils in commonly consumed food matrices would also be effective in reducing PPBG.

## Figures and Tables

**Table 1:** Formulations for study foods prepared with lentil puree or potato.

Name of the Meal	Potato Flakes/Lentil Puree		Other Ingredients		Water		Total	Cooking Procedure
	g	%	g	%	g	%	g	
Study foods containing potato; 50 g avCHO								
Apricot Chicken	136.2	11.1	351.2	28.6	742.0	60.4	1229.4	Bake at 180 °C for ~50 min (IT 70 °C)
Chicken Pot Pie	68.0	6.0	688.7	61.1	370.0	32.8	1126.7	Bake at 200 °C for ~20 min (IT 65 °C)
Lemony Parsley Soup	68.0	6.9	194.3	19.8	720.0	73.3	982.3	Simmer ingredients for ~20 min and add potato flakes and water
Potato Soup	68.0	10.9	39.2	6.3	515.8	82.8	623.0	Simmer ingredients for 30 min and add potato flakes and water
Vegetable Casserole	46.0	8.2	393.4	70.3	120.0	21.5	559.4	Bake at 180 °C for ~30 min (IT 70 °C)
Minestrone Casserole	68.0	11.4	107.6	18.1	418.4	70.4	594.0	Simmer ingredients for 40 min, add potato flakes and water; bake at 180 °C for ~25 min (IT 65 °C)
Shepherd’s Pie	68.0	6.5	615.1	58.4	370.0	35.1	1053.1	Bake at 1800 °C for ~60 min (IT 75 °C)
Vegetarian Meatloaf	68.0	9.5	274.2	38.5	370.0	52.0	712.2	Bake at 180 °F for ~30 min (IT 70 °C)
Study foods containing lentil puree; 25 g avCHO								
Apricot Chicken	321.0	48.0	347.1	52.0	0.0	0.0	668.1	Bake at 180 °C for ~50 min (IT 70 °C)
Chicken Pot Pie	321.0	31.9	686.8	68.1	0.0	0.0	1007.8	Bake at 200 °C for ~20 min (IT 65 °C)
Lemony Parsley Soup	321.0	37.2	192.3	22.3	350.0	40.5	863.3	Simmer ingredients for ~20 min
Lentil Soup	321.0	63.4	39.5	7.8	145.5	28.8	506.0	Simmer ingredients for 30 min and add pureed lentils
Side Dish	321.0	77.5	93.3	22.5	0.0	0.0	414.3	After sautéing onion and garlic add cooked lentils and heat to 65 °C
Minestrone Casserole	321.0	67.3	108.3	22.7	47.7	10.0	477.0	Simmer ingredients for 40 min, add lentils; bake at 180 °C for ~25 min (IT 65°C)
Meatloaf	321.0	36.0	570.2	64.0	0.0	0.0	891.2	Bake at 180 °C for ~60 min (IT 75 °C)
Vegetarian Meatloaf	321.0	54.4	269.5	45.6	0.0	0.0	590.5	Bake at 180 °C for ~30 min (IT 70 °C)

avCHO, available carbohydrate; IT, internal temperature.

**Table 2:** Nutritional composition of whole lentil products and measures of glycemic response.

Food	Food Code	Portion (g)	Ash (g)	Fat (g)	Energy (kcal)	Protein (g)	TCHO * (g)	TDF (g)	Moisture (g)	Palatability Score	Area Under Glucose Curve (iAUC)	Relative Glycemic Response (RGR)	Glycemic Index (GI)
Boiled Cooked Lentil	BWL	321.8	2.7	1.4	402	25.3	72.1	22.0	230	58 ± 9 ^ab^	65 ± 10 ^c^	36 ± 5 ^c^	25 ± 3 ^c^
Boiled Lentil Puree	BLP	365.3	2.6	1.0	385	24.6	70.1	20.1	277	58 ± 11 ^ab^	68 ± 11 ^c^	38 ± 5 ^c^	27 ± 4 ^c^
Frozen Cooked Lentil Puree	FLP	346.7	2.6	1.1	387	24.5	69.5	19.4	259	47 ± 10 ^abc^	113 ± 17 ^bc^	61 ± 8 ^c^	44 ± 6 ^c^
Roasted Lentil Flour	RLF	113.0	2.9	1.7	414	27.0	72.6	22.6	319	21 ± 7 ^c^	87 ± 13 ^c^	53 ± 11 ^c^	38 ± 8 ^c^
Spray-Dried Lentil Flour	DLF	100.7	2.3	1.7	377	24.9	65.7	15.7	306	30 ± 8 ^bc^	170 ± 23 ^b^	93 ± 9 ^b^	66 ± 6 ^b^
Instant Potato Flakes	IPF	68.1	2.0	0.6	244	5.0	54.6	4.6	388	50 ± 8 ^ab^	268 ± 40 ^a^	144 ± 12 ^a^	102 ± 8 ^a^
White Bread	WB	108.0	1.1	0.8	247	8.8	50.4	2.5	49	67 ± 9 ^a^	192 ± 22 ^d^	100	71

kcal, Calories; TCHO, total carbohydrate; TDF, total dietary fiber; avCHO, available carbohydrate. * Available carbohydrates determined by difference, avCHO = TCHO − TDF. The composition of all foods was determined from proximate and dietary fiber analysis obtained commercially (Maxxam Analytics International Corporation, Mississauga, ON, Canada). RGR, glycemic response relative to white bread; iAUC, area under the blood glucose response curve; GI, Glycemic Index. For iAUC, RGR, and GI, values are mean ± SEM for *n* = 10 participants. Values in the same column with different letter superscripts differ significantly (*p* < 0.05).

**Table 3:** Nutritional composition of food items and measures of glycemic response.

Food	Food Code	Portion (g)	Ash (g)	Fat (g)	Energy (kcal)	Protein (g)	TCHO * (g)	TDF (g)	Moisture (g)	Area Under Curve (iAUC)	Relative Glycemic Response (RGR)
**Study foods containing pureed lentils; 25 g avCHO**											
Apricot Chicken	AC	168.9	2.0	5.9	274	21.4	33.6	8.6	106	53 ± 8 ^a^	49 ± 12 ^bcd^
Chicken Pot Pie	CPP	266.0	3.2	3.9	311	30.4	38.6	13.6	190	44 ± 7 ^ab^	39 ± 8 ^cd^
Lemony Parsley Soup	LPS	423.7	3.0	3.1	284	16.3	48.3	23.3	353	60 ± 9 ^a^	49 ± 5 ^bcd^
Lentil Soup	S	304.9	2.7	2.3	271	18.2	44.5	19.5	237	58 ± 12 ^a^	46 ± 7 ^bcd^
Side Dish	SD	179.9	1.3	15.4	344	14.0	37.2	12.2	112	21 ± 7 ^b^	16 ± 5 ^e^
Minestrone Casserole	MC	210.1	1.3	4.1	265	18.6	38.4	13.4	148	42 ± 9 ^ab^	31 ± 5 ^d^
Meatloaf	ML	219.3	2.2	19.9	421	26.8	33.8	8.8	137	49 ± 8 ^a^	44 ± 10 ^bcd^
Vegetarian Meatloaf	VM	161.3	2.6	5.0	248	17.0	33.7	8.7	103	60 ± 12 ^a^	49 ± 8 ^bcd^
**Study foods containing potato; 50 g avCHO**											
Apricot Chicken	pAC	413.0	2.5	7.8	364	17.9	55.4	5.4	330	130 ± 18 ^abc^	70 ± 8 ^abcd^
Chicken Pot Pie	pCPP	658.0	7.2	7.7	559	65.9	55.9	5.9	521	111 ± 13 ^c^	62 ± 7 ^bcd^
Lemony Parsley Soup	pLPS	704.0	4.9	3.9	310	7.0	62.0	12.0	626	165 ± 25 ^ab^	87 ± 10 ^ab^
Potato Soup	pS	575.0	4.0	2.9	293	6.7	60.3	10.3	501	177 ± 18 ^a^	102 ± 14 ^a^
Vegetable Casserole	pSD	481.0	3.4	10.0	365	9.5	59.1	9.1	399.0	125 ± 20 ^bc^	65 ± 5 ^bcd^
Minestrone Casserole	pMC	439.0	2.6	3.2	307	13.4	56.1	6.1	363.6	163 ± 29 ^ab^	84 ± 7 ^abc^
Sheppard’s Pie	pSP	459.0	4.1	32.6	661	34.9	56.9	6.9	330.3	93 ± 14 ^c^	47 ± 3 ^bcd^
Vegetarian Meatloaf	pVM	403.0	5.2	11.5	436	23.2	59.7	9.7	303.6	126 ± 27 ^bc^	64 ± 9 ^bcd^

kcal, Calories; TCHO, total carbohydrate; TDF, total dietary fiber; avCHO, available carbohydrate. * Available carbohydrates determined by difference, avCHO = TCHO − TDF. The composition of all foods was determined from proximate and dietary fiber analysis obtained commercially (Maxxam Analytics International Corporation, Mississauga, ON, Canada). RGR, glycemic response relative to white bread; iAUC, area under the blood glucose response curve; GI, Glycemic Index. For iAUC and RGR values are mean ± SEM for *n* = 10 participants. Values for iAUC within each group of study foods with different letter superscripts differ significantly (*p* < 0.01). Values for RGR in both study groups with different letter superscripts differ significantly (*p* < 0.01).

**Table 4:** Four-way ANOVA for Consumer (C) *n* = 92; Formulation (F) *n* = 6; Gender (G) *n* = 2; Age Group (A) *n* = 3 for acceptability of lentil formulations with mean values for formulation and age group.

	Source of Variation (*F*-Value)						Mean Acceptability for Formulation ^1^						Mean Acceptability for Age Group		
Attribute	C	F	G	A	F*G	F*A	ML	MC	AC	CPP	LPS	LS	18–24 years; *n* = 27	25–34 years; *n* = 30	35 years and over; *n* = 35
Aroma ^2^	1.53 **^4^	7.64 ***	1.07 NS	0.99 NS	†	†	6.3 (1.7) ^ab^	5.4 (1.6) ^d^	6.4 (1.8) ^ab^	5.8 (1.7) ^cd^	6.7 (1.7) ^a^	6.2 (1.4) ^bc^	6.0 (1.7) ^a^	6.1 (1.7) ^a^	6.3 (1.6) ^a^
Appearance ^2^	3.40 ***	5.70 ***	1.03 NS	6.39 **	2.49 *	†	5.5 (1.8) ^bc^	5.3 (1.7) ^bc^	5.6 (1.8) ^b^	5.2 (1.9) ^c^	6.4 (1.9) ^a^	5.5 (1.8) ^bc^	5.0 (1.8) ^b^	5.6 (1.8) ^a^	6.0 (1.8) ^a^
Flavor ^2^	1.68 ***	15.95 ***	1.54 NS	4.16 **	†	†	5.8 (2.1) ^b^	5.1 (1.9) ^c^	7.0 (1.6) ^a^	6.6 (1.6) ^a^	6.6 (1.7) ^a^	5.9 (1.7) ^b^	5.9 (1.8) ^b^	6.1 (1.8) ^ab^	6.5 (1.9) ^a^
Texture ^2^	2.14 ***	9.55 ***	0.72 NS	2.49 NS	†	†	5.9 (2.0) ^cd^	5.6 (1.8) ^d^	7.0 (1.4) ^a^	6.2 (2.0) ^bc^	6.6 (1.6) ^ab^	5.8 (1.8) ^d^	5.9 (1.8) ^a^	6.2 (1.8) ^a^	6.4 (1.9) ^a^
Overall Acceptability ^2^	1.82 ***	13.20 ***	1.66 NS	2.26 NS	†	†	5.8 (2.1) ^b^	5.0 (1.7) ^c^	6.8 (1.7) ^a^	6.4 (1.8) ^a^	6.4 (1.9) ^a^	5.7 (1.6) ^b^	5.8 (1.8) ^a^	6.0 (1.8) ^a^	6.2 (2.0) ^a^
FACT ^3^	2.02 ***	13.79 ***	3.41 NS	4.78 **	†	†	4.9 (2.1) ^cd^	4.1 (1.7) ^e^	5.9 (1.8) ^a^	5.3 (1.8) ^bc^	5.4 (1.9) ^b^	4.7 (1.7)**^d^**	4.7 (1.7) ^b^	4.9 (1.9) ^b^	5.4 (2.1) ^a^

^1^ ML, Meatloaf; MC, Minestrone Casserole; AC, Apricot Chicken; CPP, Chicken Pot Pie; LPS, Lemony Parsley Soup; LS, Lentil Soup; FACT, food action rating scale. † sums of squares pooled with the error term as the probability of the effect was >0.05. ^2^ 9 = like extremely; 8 = like very much; 7 = like moderately; 6 = like slightly; 5 = neither like nor dislike; 4 = dislike slightly; 3 = dislike moderately; 2 = dislike very much; 1 = dislike extremely. ^3^ 9 = I would eat this every opportunity I had; 8 = I would eat this very often; 7 = I would frequently eat this; 6 = I like this and would eat it now and then; 5 = I would eat this if available but would not go out of my way; 4 = I do not like this but would eat it on an occasion; 3 = I would hardly ever eat this; 2 = I would eat this if there were no other food choices; 1 = I would eat this only if forced. ^4^ * *p* < 0.05; ** *p* < 0.01; *** *p* < 0.001; NS, not significant at *p* ≥ 0.05. ^abcde^ Mean values (followed in brackets by the standard deviation) within the same variable of “Formulation” and “Age Group” with the same letter within the same row (attribute) are not significantly different (*p* ≥ 0.05).
